# Emotion in Chinese Words Could Not Be Extracted in Continuous Flash Suppression

**DOI:** 10.3389/fnhum.2019.00309

**Published:** 2019-09-12

**Authors:** Kaiwen Cheng, Aolin Ding, Lianfang Jiang, Han Tian, Hongmei Yan

**Affiliations:** ^1^MOE Key Lab for Neuroinformation, University of Electronic Science and Technology of China, Chengdu, China; ^2^School of Foreign Languages, Southwest Jiaotong University, Chengdu, China

**Keywords:** emotion, simplified Chinese, breaking continuous flash suppression, conscious awareness, preconsciousness

## Abstract

Previous studies have demonstrated the automatic vigilance effect for faces and pictures and have attributed it to the brain’s prioritized unconscious evaluation of early evolutionary stimuli that are critical to survival. Whether this effect exists for evolutionarily more recent stimuli, such as written words, has become the center of much debate. Apparently contradicting results have been reported in different languages, such as Hebrew, English, and Traditional Chinese (TC), with regard to the unconscious processing of emotional words in breaking continuous flash suppression (b-CFS). Our current study used two experiments (with two-character words or single-character words) to verify whether the emotional valence or the length of Simplified Chinese (SC) words would modulate conscious access in b-CFS. We failed to replicate the findings reported in Yang and Yeh (2011) using TC, but found that complex high-level emotional information could not be extracted from interocularly suppressed words regardless of their length. Our findings comply with the distinction between subliminal and preconscious states in Global Neuronal Workspace Theory and support the current notion that preconsciousness or partial awareness may be indispensable for high-level cognitive tasks such as reading comprehension.

## Introduction

Emotional information is important for human survival, and many researchers have investigated whether emotion processing in the human brain is automatic. Previous studies have demonstrated privileged emotional processing for faces and pictures, even without the observers’ conscious awareness. In particular, the quicker detection of masked negative faces and fear-relevant natural categories such as snakes or spiders has been interpreted as the automatic vigilance effect, in which negative information draws attention automatically and is evaluated more rapidly than neutral or positive information (Estes and Adelman, 2008). This effect might occur due to the existence of a fast subcortical pathway to visually process evolution-relevant stimuli (Whalen et al., 2004; Johnson, 2005; Almeida et al., 2008; Fang et al., 2016). However, it is also well known that the evaluation of symbolic stimuli such as written words cannot be subserved by the most-cited evolutionary subcortical pathway but instead requires extensive cortical processing *via* the ventral visual pathway underlying lexical and semantic access (Cohen and Dehaene, 2004). Thus, whether emotional information can be extracted from written words in the absence of conscious awareness has become the center of much controversy. On the one hand, evidence on unconscious emotional or semantic processing has mostly been obtained from conventional blinding paradigms such as masking, binocular rivalry, and attentional blink (Dehaene et al., 1998; Gaillard et al., 2006; Van Gaal et al., 2014; Zhang et al., 2014). On the other hand, doubts have also been raised regarding the use of these paradigms, in which participants may be partially aware of some low-level perceptual features, as these paradigms highly depend on short durations to present invisible stimuli successfully (Kouider and Dupoux, 2004; Kouider and Dehaene, 2007).

The continuous flash suppression (CFS) paradigm is known to entail deeper and longer suppression of stimuli, establishing objective unawareness in which unconscious processing might produce robust behavioral and neurophysiological effects (Tsuchiya and Koch, 2005; Jiang et al., 2007). Moreover, as a variant of the CFS paradigm, breaking continuous flash suppression (b-CFS; Stein et al., 2011) has been increasingly used to investigate the extent to which emotional stimuli are processed without conscious awareness. In a typical b-CFS setting, a critical stimulus (usually at low contrast) is presented in one eye, while a continuous flashing stream of high-contrast dynamic Mondrian pictures is presented to the other eye, initially dominating an observer’s awareness. The contrast of the static stimulus is then gradually increased until it breaches CFS suppression. The time of release from suppression is commonly used as an index of the unconscious processing of the stimulus (Gayet et al., 2014; Hung et al., 2016; Rabovsky et al., 2016; Nakamura and Kawabata, 2018). Many researchers have utilized the b-CFS paradigm and have found shorter release times for invisible fearful faces than for neutral ones (Yang et al., 2007; Gray et al., 2013; Stein et al., 2014). Likewise, if words with a negative valence are released from CFS suppression faster, on average, than neutral words, some kind of unconscious emotional processing is expected to have occurred (Jiang et al., 2007). However, upon reviewing the literature, conflicting findings have been reported regarding the unconscious processing of emotional information in words when suppressed through b-CFS. Yang and Yeh (2011) demonstrated that compared to neutral words [two-character Traditional Chinese (TC) compounds, e.g., “
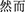
”], words describing a negative emotion, or emotion-label words (e.g., “
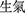
” or “anger”), and words inducing a negative emotion, or emotion-laden words (e.g., “
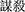
” or “murder”), required a longer amount of time to break from suppression. However, Sklar et al. (2012) found that Hebrew phrases with negative emotional valence (e.g., eternal rest) yielded shorter suppression durations than those with neutral emotion valence (e.g., dining table), even though each part (e.g., eternal or rest) of the negative phrase is in itself neutral (negative valence could not be obtained unless parts were integrated into a whole). Seemingly, both results suggested that emotion could be extracted through such symbolic stimuli as written words or phrases even if the stimuli were perceived as being invisible due to strong interocular suppression. Actually, these two studies obtained apparently contradictory results: Yang and Yeh (2011) reported that negative words took longer to enter consciousness, while Sklar et al. (2012) indicated that negative words took less time than neutral words. Most recently, Rabagliati et al. (2018) failed to replicate either of these studies using English words and phrases with an experimental design they claimed had greater power and with more proper data analysis methods (both raw and log-transformed). They upheld that there was no automatic vigilance effect on suppression times for language. Thus, further study regarding high-level emotional influences on b-CFS are highly desirable. Here, we postulate that language type might play a role in deciding whether emotion can be extracted under b-CFS. It is likely that Chinese words are more susceptible to unconscious semantic processing than English words because they are more iconic and less arbitrary (Rabagliati et al., 2018). The Chinese logographic writing system (the fertile visual structure of each character) is generally known for its morphology mapping closer to meaning than phonology, compared with alphabetic languages such as English (Wang, 1973). Therefore, its close orthographic–semantic relationship may facilitate unconscious semantic processing through “unconscious binding” process in which the unconscious mind not only encodes individual low-level features but also combines distributed features to induce a high-level representation, such as word form and meaning (Lin and He, 2009; Yang and Yeh, 2011). Coupled with the subcortical pathway for emotion, we posit that emotion could be easier to extract from Chinese characters during CFS.

To address this issue, the present study aims to replicate Yang and Yeh’s (2011) results using a different version of Chinese [Simplified Chinese (SC) characters] and a higher-powered design. First, just as spoken Chinese has a variety of dialects, such as Mandarin and Cantonese, there are two types of the written form – TC and SC. Although TC prevails in Taiwan and Hong Kong, SC, as the only official language of mainland China, is designed to simplify character structural complexity and reduce the total number of Chinese characters. This simplification covers approximately 40% of TC characters and is mainly a reduction in the number of strokes (Tsai et al., 2012). Due to the difference between the two types of written Chinese, the unconscious emotion extraction reported by Yang and Yeh (2011), who used TC, might not be significant for SC. Second, there are some possible drawbacks to the design of Yang and Yeh’s experiments. For example, using a small set of stimuli and having too many repetitions of each word could make a memorization effect very plausible due to the closer orthographic–semantic mapping inherent in the Chinese writing system (Yang and Yeh, 2011).

The second goal of this study is to gauge whether and how words presented closer to the fovea may affect unconscious emotional processing. Rabagliati et al. (2018) hypothesized that the difference in the results between studies using Hebrew and English might be the length of the stimuli because the phrases used in Sklar et al. (2012) were typically shorter (in terms of the number of characters) than those used in their study. They crossed the semantic manipulation with word length in one experiment: words were either short (3 or 4 letters) or long (7–12 letters). Suppression times were reduced for long words than short words, but not affected by the valence of those words. They concluded that stimuli presented closer to the fovea might result in a clearer perception but would not make a difference to subliminal emotional extraction. Here, we attempt to test whether the length of Chinese words affects emotional extraction under similar circumstances. We argue that one-character words (approximately three letters in English) will be closer to the fovea and will be perceived more clearly, such that the valence of one-character words may contribute more to differentiating breakthrough times than two-character words (approximately seven letters in English).

In short, to assess the replicability of the findings reported in the abovementioned work by Yang and Yeh (2011) and to determine the influence of the distance between stimuli and the fovea on unconscious emotional processing, we will investigate whether negative words written in SC break continuous flash suppression faster than neutral words, suggesting an automatic vigilance effect, using two experiments (two-character words and single-character words for Experiments 1 and 2, respectively). We believe that the findings of a replication study will be of greater significance than many “one-off” findings, since the available results from the relevant CFS literature are controversial (Moors et al., 2019).

## Experiment 1

As mentioned in the *Introduction*, the rationale for b-CFS experiments is that differences in suppression times between conditions can reveal differences in the processing of stimuli prior to conscious awareness. Following Experiment 1 of Yang and Yeh (2011), we used two-character SC words with a negative valence (i.e., emotion-label words) as the critical stimulus and neutral words as the counterpart to maximize the difference in the suppression times between emotional words and non-emotional words. Second, to guarantee that differences in suppression times were caused by high-level processing (semantic or emotional), we manipulated the word form (upright or inverted) to disrupt high-level processing. As the physical properties of upright and inverted words were identical, the difference in the detection times was assumed to reflect the sensitivity or familiarity of the global structure involved in access to awareness.

**FIGURE 1:**
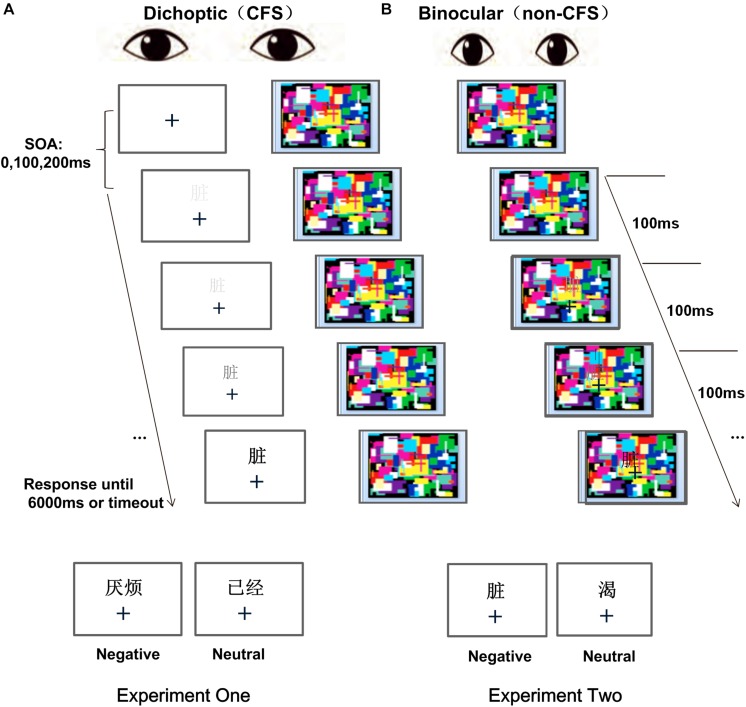
Schematic diagram of a CFS session **(A)** and a non-CFS session **(B)** in Experiments 1 and 2. In each trial, a two-character (single-character) word was presented with a ramped up contrast, and participants were asked to press the “Z” key as soon as any part of the word became visible and then to judge whether the suppressed word was above or below the fixation by pressing O or K, respectively.

Another way to ensure unconscious processing is to combine objective discrimination performance with a direct measure of subjective awareness, such as participants’ confidence ratings or visibility reports (Stein and Sterzer, 2014). Here, we adopted a visibility test to serve as a criterion for data exclusion and participant screening. Our participants completed a visibility test between sessions (between CFS and non-CFS sessions, see Figure 1) rather than between each trial, as we thought trial-by-trial insertion would interrupt participants’ engagement with the task, potentially impairing the processing of stimuli during the experiment (Rabagliati et al., 2018).

This experiment followed a 2 (emotional valence: negative, neutral) × 2 (word form: upright, inverted) within-subjects factorial design. Each of the four conditions contained 80 trials, and each participant completed two blocks for a total of 320 trials in a dichoptic session (CFS). The eye to which the Mondrian images or the word was projected in the CFS session was counterbalanced across the two blocks. The presentation of stimuli and their location relative to the fixation cross were also randomized. We set different stimulus onset asynchronies (SOAs) between the onset time of the first Mondrian mask and that of the word (0, 100, or 200 ms) to add uncertainty as to when the target would appear.

Following relevant studies using CFS (Jiang et al., 2007; Costello et al., 2009; Stein et al., 2011; Yang and Yeh, 2011; Nakamura and Kawabata, 2018), we also implemented a binocular control experiment (non-CFS session) to ensure that the obtained results were caused by processing during, but not after, interocular suppression. In the non-CFS session, the word was superimposed on the dynamic Mondrian pictures and was presented to both eyes. The contrast of the word was gradually increased from 0 to 50% within 3.3 s and was kept constant afterward. The timing was the same as that in Yang and Yeh (2011) to minimize differences in detection times between the two sessions. The other manipulations were the same as those in the CFS session, except that the total number of trials was cut in half (160 trials). Thus, it is plausible that the confounding effects among all conditions were likely to be the same for a participant across two sessions, and the critical difference between the sessions was whether the stimulus was perceived with or without conscious awareness. Presumably, if there was a difference in RTs between negative and neutral words or between upright words and inverted words in the dichoptic session but not in the binocular session, this difference might be due to different processing mechanisms in the unconscious state. In contrast, if the results were similar in both binocular and dichoptic sessions, they might be caused by postperception differences, such as setting different criteria for the different types of words in the conscious state.

### Materials and Methods

#### Participants

Twenty-two participants (11 males and 11 females), aged 22–27 years old (average 23.4 ± 1.3), were recruited to participate in our two-session experiment, which lasted approximately 1 h, with the dichoptic session lasting 40 min and the binocular session lasting 20 min. All participants were native Chinese speakers and were skilled readers of SC from Mainland China, with normal or corrected-to-normal vision, and no history of psychiatric disorders. Participants were naïve to the purpose of the experiment and gave their written informed consent beforehand. Each participant was given $8 an hour as financial compensation. The experimental paradigm was approved by the Ethics and Human Participants in Research Committee at the University of Electronic Science and Technology of China. All research was conducted in accordance with the Declaration of Helsinki.

#### Apparatus

Visual stimuli were presented on a 21-inch color monitor with a mean luminance of approximately 22 cd/m^2^ and a frame frequency of 100 Hz at a spatial resolution of 1,280 × 1,024 pixels. The experimental program was compiled by MATLAB 2013b with Psychtoolbox (Brainard, 1997). In the dichoptic phase, participants viewed the stimuli (Font: Courier New, Size: 25) against a gray background *via* a mirror stereoscope in a sound-attenuated room. The stereoscope was composed of two intermediate mirrors (angled ± 45° orthogonally) and the other two adjustable outer mirrors. A chin rest was used to prevent head movements. The mirror stereoscope and the chin rest were positioned 57 cm from the monitor, with a vertical cardboard divider splitting the display so that each eye only saw half of the screen. Participants used the “Z,” “O,” and “K” keys on a standard keyboard to respond as the trials proceeded. To promote stable binocular fusion, the stereoscope was adjusted for each participant.

#### Stimuli

Following Yang and Yeh (2011), we used a set of 80 Chinese two-character compound words that contained an equal number of negative (e.g., “
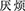
” or “annoying”) and neutral words (“
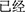
” or “already”). It is notable that functional words are used as neutral words instead of content words to achieve the maximal difference in the latency between emotional and non-emotional words breaching interocular suppression. Here, emotional words were specified as emotion-label words (e.g., “
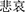
” or “sad,” “
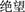
” or “despairing”). Based on the results of questionnaires delivered to 64 volunteers, we used a seven-point Likert scale to control for emotional valence [neutral, 4.82 ± 0.20; negative, 3.26 ± 0.29, *t*(39) = –31.27, *p* = 0.000] and arousal [neutral, 3.43 ± 0.44; negative, 5.13 ± 0.31, *t*(39) = –19.81, *p* = 0.000]. These two kinds of words were also matched on stroke (the mean stroke count: neutral, 15.98; negative, 16.47) and word frequency (the mean frequency per million words: neutral, 40.27; negative, 39.64) (Language Teaching, and Research Institute of Beijing Language Institute, 1986). Each two-character word extended 1.5° × 0.75° and was presented 1.27° (center-to-center) above or below the fixation (a cross, 0.8° × 0.8°) with equal probability. In inverted conditions, each word was virtually mirror inverted to remove high-level information and preserve low-level local features in the space domain.

#### Procedure

Before the experiment, participants underwent a simple test for eye dominance (Porta, 1593). Five out of 22 participants were classified as left-eye dominant. Afterward, participants performed 10–50 practice trials to ensure that the mirror stereoscope was properly adjusted to a comfortable position and that they were familiarized with the procedure. Those whose accuracy exceeded 90% were allowed to continue with the formal two-session experiment. Figure 1 shows a schematic illustration of one trial in this experiment.

Each trial began with two symmetrical squares (10.70° × 10.70° visual angle, with a thickness of 0.2°) against a gray background. Both squares were surrounded by a frame with a fixation cross at the center to promote the convergence of the two images in the participants’ perception. Participants were instructed to focus on the fixation cross with both eyes open, without blinking or diverting their attention. After participants pressed “Z,” the fixation cross remained superimposed on both screens, and a dynamic Mondrian mask was presented to one eye. After 0, 100, or 200 ms, a Chinese compound (two-character) word was presented to the other eye slightly above or below the fixation cross, and its level of contrast was continuously ramped up from 0 to 50% over 500 ms and was then kept constant until the end of the trial (the ramping-up procedure ensured that the target was suppressed by the CFS mask when it was initially presented). The Mondrian mask (extended 5.5° × 5.5°) consisted of a set of flashing squares that randomly changed in size, color, contrast, rotation, and position at a rate of 10 Hz. Participants were instructed to press “Z” as quickly as possible once they detected any part of the word and then to proceed to judge the location of the word. Both RTs (the time from the onset of the word to the button press) and accuracy were recorded by the computer. The trial ended immediately at button press or if no response was made after 6 s. Participants were given the choice to take a short break after every 40 trials or between every two blocks.

Immediately after two blocks, participants were presented with a subjective visibility test that consisted of 80 words, half of which were included in preceding stimuli. The participants were asked to decide which of these words they had seen in the preceding experiment without time pressure. In this two-choice visibility test, the mean accuracy (SD) was 34.3% (17.3%), far below the level of chance at 50% [*t*(21) = –4.17, *p* = 0.00, two-tailed], which confirmed that the majority of participants (only one beyond 60%) were unable to consciously perceive the content of the suppressed word.

#### Analysis and Results

**FIGURE 2:**
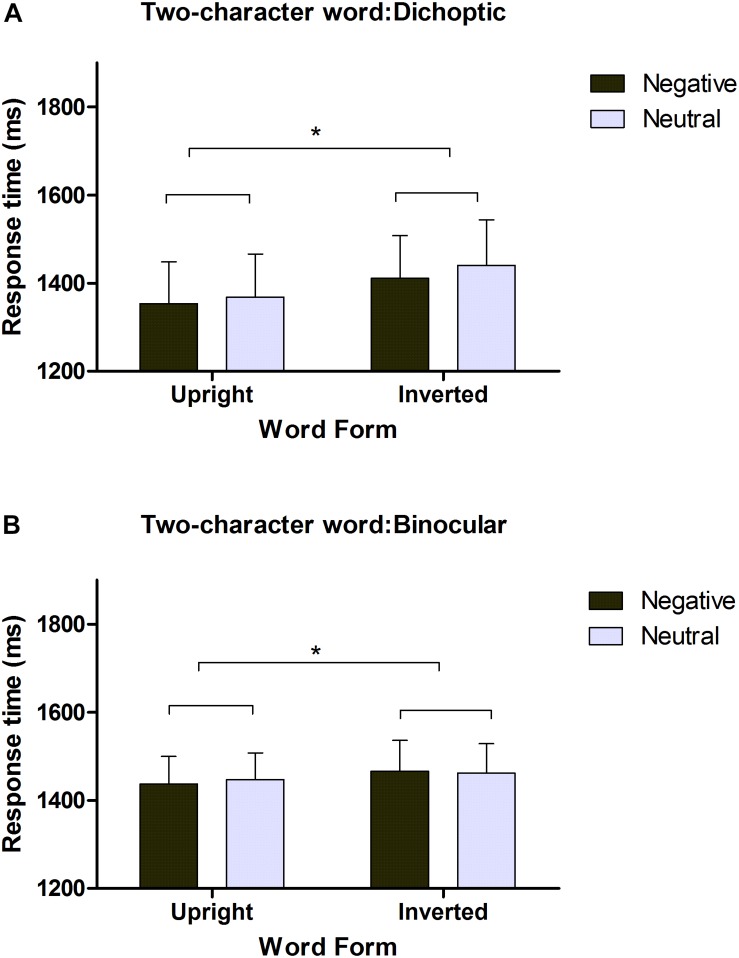
Reaction times (RTs) of negative and neutral words in a dichoptic session **(A)** and a binocular session **(B)**. Error bars denote SEM (*n* = 20). ^∗^*p* < 0.05.

One participant did not complete the first session due to the suppression effect being too strong, and another participant achieved accuracy higher than 60% in the subjective visibility test, implying that his responses might have been contaminated by partial awareness in the dichoptic session; therefore, these two participants were excluded from further analysis. For the remaining 20 participants, response times (RTs) were calculated based on correct trials only, and very few trials were excluded because the mean correct response rate was quite high (94%). We also excluded outlier trials in which the RT was longer than 6 s (the timeout) or shorter than 500 ms and other trials in which the RT was more than three standard deviations from the sample mean. We excluded these trials because if the target was not able to be detected within 6 s or appeared to be detected abnormally quickly (compared to the sample mean), the observed RT could reflect unknown factors (e.g., inattention or involuntary button press). Overall, fewer than 5% of the correct trials were removed from our analysis based on these criteria. The RTs were submitted to 2 (emotional valence: negative, neutral) × 2 (word form: upright, inverted) repeated-measures analysis of variance (ANOVA), and the results are shown in Figure 2A. Based on G^∗^ Power 3.1.9.4, we achieved a power larger than 0.95 at an α level of 0.05, with the smallest effect size (η^2^ = 0.198) in our ANOVAs (Faul et al., 2007).

In the dichoptic session, the main effect of emotion was not significant [M neutral = 1,404 ms, M negative = 1,382 ms, *F*(1,19) = 2.156, *p* > 0.05] but the main effect of word form was significant [M upright = 1,361 ms, M inverted = 1,426 ms, *F*(1,19) = 5.975, *p* = 0.024, partial η^2^ = 0.239]. Comparative analysis showed that upright words were detected 65 ms faster than inverted words. There was no interaction between emotion and word form [*F*(1, 19) = 0.122, *p* > 0.05]. Since some researchers hypothesized that the suppression would be much deeper when the participant’s dominant eye was masked, we selected trials in which words were presented to the non-dominant eye (Moors et al., 2017b; Yang et al., 2010). Additional analysis of the data in selected trials reinforced the aforementioned results. No significant difference was found between neutral and negative words [M neutral = 1,282 ms, M negative = 1,265 ms, *F*(1, 19) = 0.847, *p* > 0.05], while significant differences were found between upright and inverted words [M upright = 1,247 ms, M inverted = 1,300 ms, *F*(1,19) = 5.364, *p* = 0.032, partial η^2^ = 0.220]. Comparative analysis showed that words breached suppression 53 ms faster when they were upright than when they were upside down. There was no interaction between emotion and word form [*F*(1, 19) = 0.01, *p* > 0.05].

In the binocular session (non-CFS), the same criteria were employed to exclude outliers, and very few trials were excluded because the mean accuracy rate was high (96.5%). As shown in Figure 2B, the main effect of emotion was not significant [M neutral = 1,461 ms, M negative = 1,454 ms, *F*(1, 19) = 0.140, *p* > 0.05]. The main effect of word form was significant [M upright = 1,442 ms, M inverted = 1,464 ms, *F*(1, 19) = 4.704, *p* = 0.043, partial η^2^ = 0.198]. Comparative analysis showed that upright words could break suppression 22 ms faster than inverted words in residual or partial awareness. There was no interaction between emotion and word form [*F*(1, 19) = 0.584, *p* > 0.05].

#### Discussion

The results of the dichoptic session in this experiment were at odds with the results in Yang and Yeh (2011), in which more time was needed to detect negative words than neutral words when the words were upright but not when they were inverted. In our experiment, negative and neutral two-character words were detected at almost the same speed whether they were upright or inverted. Furthermore, statistics for the trials in which each participant’s dominant eye was suppressed reinforced the above findings. As far as word form was concerned, upright words were detected significantly faster than inverted words, from which we can speculate that low-level perceptual features such as inversion can be processed even without conscious awareness. However, this speculation is likely inaccurate because a similar pattern also occurred in the binocular viewing condition in which residual or partial awareness supposedly played a role (Kouider and Dupoux, 2004). Notably, the inversion effect was also observed in Yang and Yeh (2011) but was overlooked without any specific discussion. We remedy this drawback in our General Discussion.

## Experiment 2

In the previous experiment, we testified that there was no difference in reaction times between emotion and neutral two-character words in either CFS or non-CFS sessions. However, we still did not know whether word length would affect the detection time under the same circumstances. Thus, stimuli containing only one character in SC were used in this experiment to further examine whether emotional information *per se* could be extracted during the CFS session.

In addition, the negative words used in Experiment 1 were all emotion-label words. However, it has been well documented that emotion-label words (e.g., sadness, happiness) and emotion-laden words (e.g., death, birthday) have different cognitive and neural correlates (Altarriba, 2006; Altarriba and Basnight-Brown, 2011; Zhang et al., 2017, 2018a,b; Wang et al., 2019). Therefore, one-character emotion-laden words such as “
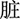
” (dirty) and “
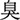
” (stink) were employed to extend or verify our results obtained using emotion-label words in Experiment 1.

### Materials and Methods

#### Participants

Another 31 participants (9 males, 22 females), aged 20–26 years old (average 23.1 ± 1.5), were recruited to participate in Experiment 2, which lasted approximately 45 min with the dichoptic session lasting 30 min and the binocular session lasting 15 min. Twenty-three of the 31 participants were classified as being left-eye dominant.

#### Apparatus, Procedure, and Design

The apparatus, procedure, and design were the same as those in Experiment 1, except that the one-character word was relocated (extended 0.75° × 0.75°) between where two-character words had been presented, as shown in Figure 1.

#### Stimuli

To control for word length, we selected two different sets of words. Sixty-eight volunteers were invited to scale the emotional valance and arousal of the words in the word bank, which consisted of 70 one-character negative words and 70 neutral words. Based on the questionnaires, we selected two sets of words (40 words for each) that were well controlled for emotional valence (neutral, 4.98 ± 0.30; negative, 2.96 ± 0.19, *t* = –79.75, *p* = 0.000) and arousal (neutral, 3 ± 0.56; negative, 5.3 ± 0.45, *t* = –17.89.75, *p* = 0.000). Negative and neutral words were also matched on stroke number (neutral, 10.5 ± 2.49; negative, 10.1 ± 2.28) and word frequency (mean frequency per million words: neutral, 33.27 ± 10.2; negative, 33.3 ± 21.4) based on the Language Teaching, and Research Institute of Beijing Language Institute (1986).

#### Analysis and Results

Two of the participants were excluded from the analysis because they had an accuracy rate higher than 60% in the subjective visibility test. RTs in both sessions were calculated based on the correct trials (correct “O” or “K” key press) of the remaining 29 participants. Using G^∗^ Power, we achieved a power larger than 0.99 at an α level of 0.05, with the smallest effect size (η^2^ = 0.316) in our ANOVAs (Faul et al., 2007).

**FIGURE 3:**
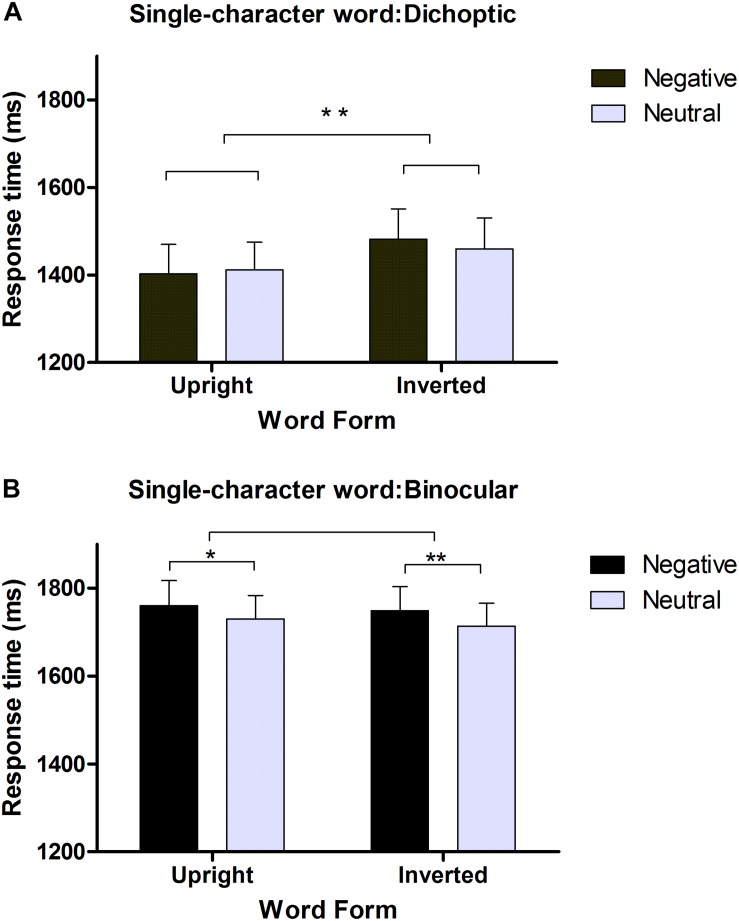
Reaction times (RTs) of negative and neutral words in a dichoptic session **(A)** and a binocular session **(B)**. Error bars denote SEM (*n* = 29). ^∗^*p* < 0.05, ^∗∗^*p* < 0.005.

In the dichoptic session, the same criteria were employed to exclude outliers as in Experiment 1. Overall, 4.1% of the trials were removed from our analysis based on these criteria. The remaining reaction times were submitted to 2 (emotional valence: negative, neutral) × 2 (word form: upright, inverted) repeated-measures ANOVA. As shown in Figure 3A, the main effect of emotion was not significant [M neutral = 1,435 ms, M negative = 1,442 ms, *F*(1, 28) = 0.357, *p* > 0.05], while the main effect of word form was significant [M upright = 1,407 ms, M inverted = 1,470 ms, *F*(1, 28) = 14.50, *p* = 0.001, partial η^2^ = 0.341]. Comparative analysis indicated that upright words broke from suppression significantly faster (63 ms) than inverted words. There was no interaction between emotion and word form [*F*(1, 28) = 2.27, *p* > 0.05]. Additionally, if we selected the RTs for each participant when the dominant eye was suppressed, similar results were obtained. No significant difference was found between neutral and negative words [M neutral = 1,506 ms, M negative = 1,518 ms, *F*(1, 28) = 0.313, *p* > 0.05], but a similar significant difference was found between upright and inverted words [M upright = 1,472 ms, M inverted = 1,551 ms, *F*(1, 28) = 12.951, *p* = 0.001, partial η^2^ = 0.316]. Comparative analysis indicated that negative words were detected faster than neutral words (M neutral = 1,479 ms, M negative = 1,466 ms), but the difference did not reach significance when words were upright. Upright words broke from suppression significantly faster (79 ms) than inverted words. There was no interaction between emotion and word form [*F*(1, 28) = 2.27, *p* > 0.05].

In the binocular session, the same criteria were employed to exclude outliers, and very few trials were excluded because the mean accuracy rate was high (97%). As shown in Figure 3B, the main effect of emotion was significant [M neutral = 1,722 ms, M negative = 1,754 ms, *F*(1, 28) = 15.276, *p* = 0.001, partial η^2^ = 0.353], while the main effect of word form was not significant [M upright = 1,745 ms, M inverted = 1,731 ms, *F*(1, 28) = 1.954, *p* > 0.05]. Comparative analysis showed that negative words were detected significantly slower (30 ms) than neutral words (M neutral = 1,730 ms, M negative = 1,760 ms) when they were upright. There was no interaction between emotion and word form [*F*(1, 28) = 0.096, *p* > 0.05].

#### Discussion

In the CFS session, even when single-character words were viewed closer to the fovea, their emotional valence did not affect suppression times (RTs). It was less likely that the null findings from Experiment 1 could be caused by the distance between the critical stimuli and the fovea or the length of words. Statistics for the trials in which each participant’s dominant eye was suppressed reinforced this observation. Moreover, compared with the binocular control session, in which negative words were detected significantly slower than neutral words, this null result could be postulated only to occur during interocular suppression rather than after suppression. Interestingly, in this experiment, the inversion effect persisted for single-character words in the dichoptic session but disappeared in the binocular control session, confirming that word form but not emotional information could be processed in the absence of conscious awareness. It is plausible that the preconscious or conscious processing of emotional information mediated the inversion effect during the binocular session.

## General Discussion

Through two higher-powered CFS studies using both two-character (Experiment 1) and single-character (Experiment 2) SC words, we identified whether emotional valence or word length affected suppression break time in the absence of conscious awareness. First, we failed to replicate the main findings reported in Yang and Yeh (2011) and found that the emotional valence of Chinese words did not modulate access to consciousness. Second, the length of words or the distance to the fovea did not affect the emotion extraction in the unconscious state, although different patterns were observed in the conscious state.

**FIGURE 4:**
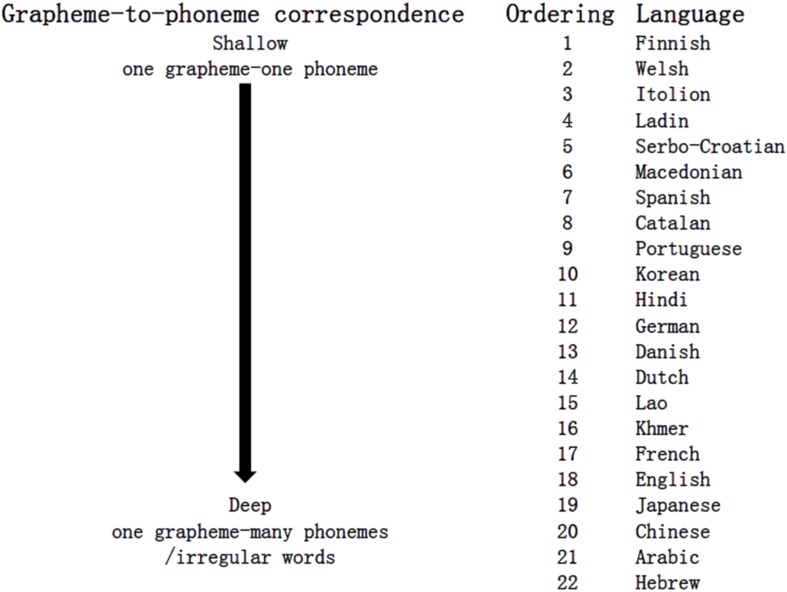
Orthographic depth of some languages (adapted from Figure 1 in Liu and Cao, 2016).

First and foremost, we controlled our stimuli based on the same criteria for emotion valence as reported in Yang and Yeh (2011) although we employed different Chinese words due to the unavailability of their stimuli in any published form [some overlap probably existed between the two lists such as “
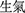
”(TC) and “
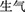
”(SC); see Appendix]. Thus, it is justifiable to discuss our findings by comparing these two studies. There are two alternative interpretations for the reason why our study produced such different results from those of Yang and Yeh’s (2011) – one being “optimistic” and the other being “pessimistic.” The optimistic interpretation is that the difference between the two versions of Chinese resulted in divergent findings across the two studies. Yang and Yeh (2011) based their findings on the hypothesis that the strokes in each Chinese character might undergo an “unconscious binding” process that contributes to a closer orthographic–semantic mapping in the brain, since TC characters evolved from ancient characters that took shape more than 3000 years ago (Lin and He, 2009; Tsai et al., 2012). They insisted that this closer orthographic–semantic mapping facilitated the extraction of emotional information from TC characters under CFS. We agreed with them on both their hypothesis and interpretation. However, SC could map less to meaning than TC due to its simplification (e.g., reductions in strokes or change in semantic parts). Virtually, the mean number of stokes in the words in our study was much smaller than that in their stimuli (neutral, 15.98 vs. 23.08; negative, 16.47 vs. 23.33). For example, the SC character “
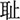
” (shameful) seems less semantically representative or emotionally valenced than the TC version “
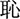
” because the “
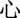
” part means heart or feeling. The same is true for “
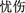
” (distressed) instead of “
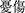
” in which “
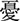
” is highly morphologically related to emotion. Thus, it is plausible that unconscious emotional processing occurred among TC speakers but disappeared in SC speakers. Furthermore, the orthographic depth hypothesis in linguistics can also be introduced to reconcile the discrepancy between our results and those of previous relevant studies (Yang and Yeh, 2011; Rabagliati et al., 2018; Sklar et al., 2018). The orthographic depth indicates how easy it is to predict the pronunciation of a word based on its spelling: shallow orthographies enable a reader to recognize printed words through their phonology, while deep orthographies encourage a reader to rely more on their visual–orthographic structures (Frost et al., 1987; Liu and Cao, 2016). The orthographic depth of various written languages can be regarded as a continuum from shallow to deep, with Chinese ranking somewhere between English (more shallow) and Hebrew (the deepest), as shown in Figure 4. As mentioned, SC, compared to TC, could be more easily recognized by its phonology than its morphology due to the simplification process (reduced structure complexity and replaced semantic parts with phonetic parts). In this sense, SC (less deep) ranks slightly closer to English, while TC (deep) ranks closer to Hebrew. It seems plausible that we obtained null findings, as in Rabagliati et al. (2018), while Yang and Yeh gained affirmative results, as in Sklar et al. (2012). Future research is needed to investigate comparisons between languages with more orthographic depth, such as Arabic or Japanese, and languages with less orthographic depth, such as Finnish or Spanish, to verify cross-linguistic differences in emotional or semantic processing related to the orthographic depth.

However, the pessimistic alternative is that defects in the experimental design might have yielded false-positive results in Yang and Yeh (2011). First, the small set of stimuli used in their experiments (24 and 32 words, respectively, in Experiment 1 and Experiment 2) make it very plausible that artificial feature bindings or false correlations existed between high-level semantic meaning and low-level features due to there being too many repetitions of each target. It has been suspected that the positive results in Yang and Yeh (2011) were not fundamentally caused by the semantics of the target words but instead caused by their low-level visual features (Rabagliati et al., 2018). Second, the small sample of participants (12 for each experiment) seemed insufficient to conduct effective data analysis using ANOVA or to produce reliable results. This flaw was aggravated by a lack of visibility measurements to exclude any participant who was thought to have partial awareness of the stimulus on some trials, producing an illusion of unconscious semantic processing (Kouider and Dupoux, 2004; Kouider and Dehaene, 2007). Given the null results for the current higher-powered experiments using more qualified participants, fewer repetitions of the stimuli, and visibility tests, we are inclined to support the pessimistic interpretation that no emotional information could be extracted from SC words when they were invisible due to strong interocular suppression. This interpretation fits well with a number of recent CFS studies on unconscious language processing in terms of word frequency (Heyman and Moors, 2014) or semantics (Kang et al., 2011; Yang et al., 2017; Rabagliati et al., 2018). In particular, Kang et al. (2011) failed to find the well-established electrophysiological marker of semantic analysis, the N400 component, when a critical word was rendered completely invisible by interocular suppression although it was preceded by semantically related primes.

Nevertheless, there was some consistency between our study and Yang and Yeh’s (2011) regarding the attempt to distinguish emotion-label words from emotion-laden words. However, no differences were observed between these two subcategories in either study despite the opposite result being observed for neutral counterparts in CFS conditions. We do not think this result contradicts ample prior evidence that emotion-label words differ from emotion-laden words either in their cognitive mechanism or in neural correlates because the b-CFS paradigm may not be as sensitive as ERPs for dissociating these two kinds of emotional words (Altarriba, 2006; Zhang et al., 2017, 2018b).

Broadly speaking, this finding seems at odds with the automatic vigilance hypothesis, suggesting that negative information in contrast to neutral or positive information receives privileged attention and quicker evaluation, even in an absence of conscious awareness. Previous evidence has demonstrated that fearful faces broke through CFS suppression quicker than neutral faces (Yang et al., 2007; Gray et al., 2013; Stein et al., 2014). It seems conceivable that the human brain is more sensitive to faces conveying fearful information than those with neural valence due to the evolutionary importance of the former to human survival in nature (Carretié, 2005; Gayet et al., 2016). According to the dual-route hypothesis of emotion evaluation, the processing of negative faces could take a subcortical “shortcut” from the thalamus to the amygdala due to the evolutionary edge (Johnson, 2005; Troiani and Schultz, 2013; Fang et al., 2016). However, symbolic carriers such as words were more recently acquired in terms of evolutionary processes, such that they could not have such an evolutionary edge over faces or naturally fearful objects. Instead, the processing of emotion words might be accomplished *via* an indirect pathway from the occipitotemporal area and the visual word form area to the amygdala (Phelps and Ledoux, 2005). Furthermore, some researchers have questioned the existence of the most-cited subcortical channel through CFS paradigms and have contended that it is not the semantic or emotional analysis of interocularly suppressed stimuli but rather the low-level visual features that affect suppression times in these seemingly effective studies (Stein et al., 2014; Hedger et al., 2015). The most thought-provoking finding was that Patient SM, who suffered complete bilateral amygdala lesion and was not able to consciously discriminate between fearful and happy faces, showed the same edge for detecting invisible fearful faces over happy faces in a CFS experiment as healthy controls (Tsuchiya et al., 2009). Hedger et al. (2015) recently found that the advantage of fearful faces breaking suppression faster than neutral faces could be predicted by the effective contrast of the stimuli (the relationship between the Fourier spectrum and the contrast sensitivity function), and Stein et al. (2014) found that a similar advantage was solely dependent on high spatial frequencies. More elusive is that this prioritization for invisible fearful faces over neutral ones persisted for inverted faces (Yang et al., 2007; Gray et al., 2013), faces with negative luminance polarity (Gray et al., 2013), eyes-only images (Yang et al., 2007), and artificial schematic face images (Stein and Sterzer, 2012). Therefore, some researchers tend to believe that the brain can only process low-level perceptual differences during CFS rather than semantic or emotional levels (Gayet et al., 2014; Moors et al., 2017a, 2019). This belief is also in accordance with a few recent studies that have questioned the evidence for the high-level processing of other kinds of stimuli rather than faces and words. These studies may potentially shed doubt on whether higher-level cognitive tasks could be accomplished during interocular suppression: numerical priming (Hesselmann and Knops, 2014; Hesselmann et al., 2015), audio-visual integration (Moors et al., 2015); scene congruency (Moors et al., 2016), and evaluative learning (Högden et al., 2018).

Methodologically speaking, no evidence of unconscious emotional or semantic processing was found in the b-CFS paradigm, but some evidence has been documented by adopting CFS in a different way such as by priming CFS (Costello et al., 2009; Eo et al., 2016). We reason that the Global Neuronal Workspace Theory, as well as the distinction between the subliminal state and the preconscious state, provides a useful framework to explain the discrepancies in the empirical evidence obtained from different paradigms (Dehaene et al., 2006; Kouider and Dehaene, 2007). Under this framework, the interactions of bidirectional (top-down and bottom-up) mechanisms determine whether preconscious high-level processing occurs successfully. The semantic priming effect (that targets preceded by visible semantically related primes break suppression quicker than those preceded by unrelated stimuli) observed in Costello et al. (2009) may have been interpreted by the effective bidirectional mechanisms in which the visible prime paves the way for the invisible target through the activation of the semantic nodes of the words. It is the feed-forward activation together with the subsequent conscious access to some low-level features such as word parts or form in CFS that results in the semantically related target becoming perceptually more prominent or detectable. This account is compatible with the findings that top-down VWM (visual working memory) modulation could bias the unconscious perceptual processing of unseen stimuli in other priming CFS paradigms (Gayet et al., 2013; Pan et al., 2014). Interestingly, Eo et al. (2016) found that a consciously presented Korean prime word affected the N400 component only when participants’ attention was diverted from a CFS-masked word. The authors maintained that reduced interocular suppression due to the diversion of attention from the target spared some top-down mental resources (e.g., VWM) to facilitate further semantic processing in the preconscious state (although the strength of mental resources is not enough to amplify the sensory information to the conscious state). This interpretation is also consistent with another recent study in which subjects who failed to judge the identity of suppressed faces showed priming effects only when a lower-level feature of the suppressed face (color or location) was visible (Gelbard-Sagiv et al., 2016). It suggested that high-level processing of face identity might be accomplished by conscious or preconscious access to low-level features. However, the b-CFS paradigm, implemented in the current study, may have been restrained in the subliminal state in which fragmented feed-forward activation produced by low-level sensory stimulation progressively died out due to a lack of top-down resources and failed to reach a higher-level workspace, resulting in no emotional (or semantic) processing (Kang et al., 2011; Moors et al., 2017a, b). In the future, we need more electrophysiological explorations to disclose the distinct temporal dynamic courses between the b-CFS and priming CFS paradigms. Concurrent neuroimaging tools should also be applied to the research topic to provide more consistent and reliable results.

Our second goal was to identify whether the length of words or the distance from the target to the fovea affects emotional extraction in Chinese. The result was that short words did not make emotional processing easier in the absence of conscious awareness. This negative finding resonated with the latest study in which suppression times were unaffected by the emotional information of English words whether the words were long or short (Rabagliati et al., 2018). In contrast, the reduction in suppression times for longer words compared to short words in their findings led to the conclusion that low-level features, such as the length of English words, could modulate conscious access. However, this conclusion could not be confirmed by our study due to the different groups of subjects recruited for the two experiments and the inappropriateness of statistically comparing two sets of data. In future work, we will cross the valence and the length of words in one experiment. Rather, we managed to obtain similar results through another low-level feature: word form. Although word form (upright or inverted) did not affect the suppression times of emotional words in the CFS condition in either of the two experiments, the word inversion effect persisted, in that upright words were found to break the suppression significantly faster than inverted words. It is noteworthy that the word inversion effect ensued in the non-CFS session in Experiment 1 but disappeared in non-CFS session of Experiment 2. This inconsistency between the two non-CFS sessions might help exclude the interpretation that preconsciousness was potentially involved in the CFS conditions. Two alternatives might account for this inconsistency. First, this result indicated that form could be processed unconsciously, echoing previous evidence regarding low-level features of targets such as color, spatial frequency, orientation, and collinearity in the CFS paradigm (Kanai et al., 2006; Bahrami et al., 2008; Willenbockel et al., 2012; Li and Li, 2015; Gelbard-Sagiv et al., 2016). In particular, this interpretation was also consistent with most previous relevant studies in which TC characters were detected faster than the same characters inversed or scrambled (Yang and Yeh, 2011, 2014), and SC words were detected faster than English or Hebrew words by Chinese readers (Jiang et al., 2007). However, one possibility cannot be ignored – that the novelty of inverted words may make them pop out more quickly than familiar objects (i.e., upright words). The difference between the two conditions cannot be solely attributed to the low-level features of the SC words. We would rather interpret this difference as the form familiarity effect (lower level compared to emotional or semantic), suggesting that upright words are more expected than inverted words because the former are more frequently encountered in daily life (Kerr et al., 2017). This interpretation is confirmed by our findings in both CFS sessions in which upright words were detected faster than inverted words despite the null findings of emotional or semantic processing. In future research, the familiarity/novelty rate should be more heavily scrutinized to improve the experimental design because familiarity might also influence emotional processing through interocular suppression. Second, it was likely that once some conscious awareness was spilt out from masking or suppression, especially in our second non-CFS session, emotional or semantic processing could have been accomplished with shorter words or words presented closer to the fovea automatically (to some extent) as the processing was task irrelevant. With limited conscious awareness, a certain amount of semantic processing could have mediated or even offset form integration or detection, resulting in the disappearance of the inversion effect in the non-CFS condition of Experiment 2. This interpretation is consistent with several electrophysiological reports showing that emotional words (positive or negative) could be separated from neutral ones, marked by a negative component (N170) in the occipito-temporal region, during attentional blink (unlike CFS, the stimuli are unattended rather than being rendered subliminal) (Zhang et al., 2014), and strong emotional processing of faces was observed in both the N170 and the LPC (late positive component) in non-CFS conditions but disappeared in CFS conditions (Schlossmacher et al., 2017).

Finally, we did not stringently control the arousal level of emotional words against neutral words in two experiments, nor did other researchers in relevant CFS studies (Yang and Yeh, 2011; Sklar et al., 2012; Rabagliati et al., 2018). According to the most recent bibliometric analysis (1990–2018), valence and arousal, the two most distinct properties of emotional words, render relatively consistent effects in that emotional words (negative or positive) with higher arousal have a processing advantage over neutral words with lower arousal, as shown in a large body of studies (Liu et al., 2019). Furthermore, for the purposes of the present work, the distinction is not critical because valence and arousal together account for the emotionality of emotional words and are both semantic attributes of the words.

## Conclusion

In sum, no unequivocal pattern has emerged from the existing studies on the unconscious emotional or semantic analysis of words. Our study questioned the generalizability of the findings reported in Yang and Yeh (2011) and obtained strong evidence for the absence of subliminal emotional processing of SC words in breaking continuous suppression (b-CFS) irrespective of the length of the words. Therefore, we may draw the conservative conclusion that high-level emotional or semantic processing is unlikely to occur in words through interocular suppression. Our findings support the distinction between subliminal and preconscious states under the global neuronal workspace theory and the current notion that awareness, or at least preconsciousness, may be indispensable for high-level cognitive tasks such as reading comprehension.

## Data Availability

All datasets generated for this study are included in the manuscript and/or the Supplementary Files.

## Ethics Statement

This study was carried out in accordance with the recommendations of “the Ethics and Human Participants in Research Committee at the University of Electronic Science and Technology of China (No. 180409)” with written informed consent from all subjects. All subjects gave written informed consent in accordance with the Declaration of Helsinki. The protocol was approved by the Ethics and Human Participants in Research Committee at the University of Electronic Science and Technology of China (No. 180109).

## Author Contributions

KC and HY conceived and designed the experiments, analyzed the data, and wrote the manuscript. KC and AD performed the experiments. LJ and HT contributed reagents, materials, and analysis tools.

## Conflict of Interest Statement

The authors declare that the research was conducted in the absence of any commercial or financial relationships that could be construed as a potential conflict of interest.

## Appendix

**Table:**

**Two-character words (Experiment 1)**				**Single-character words (Experiment 2)**			
	
	**Neutral**	**Negative**			**Neutral**	**Negative**	
Slightly			Despairing	Thirsty			Insult
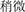
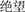
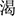
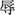
Especially			Painful	Sell			Cheat
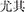
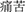
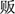
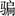
Total			Guilty	Neck			Shame
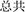
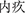
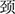
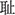
Already			Annoying	Box			Sputum
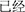
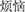
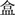
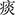
Will			Angry	Cut			Urine
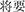
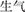
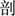
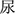
Immediate			Scared	Mask			Shit
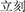
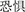
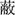
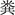
Some day			Bitter	Naked			Pus
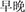
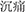
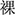
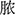
Frequently			Gloomy	Crow			Scorn
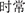
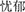
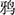
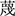
Everywhere			Anxious	Huge			Dirty
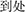
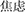
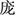
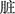
Furthermore			Sad	Fill			Basebred
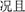
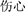
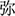
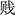
Must			Hateful	Stick			Wicked
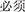
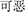
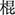
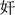
Suddenly			Resentful	Flat			Obscene
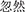
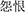
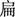
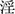
Indeed			Humbled	Hang			Curse
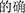
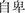
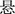
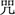
Specially			Madcap	Poor			Steal
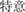
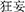
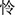
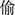
Hurriedly			Depressed	Yarn			Deceive
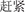
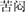
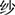
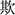
Perhaps			Downhearted	Relative			Die
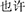
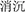
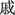
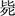
Probably			Sorry	Frame			Stink
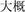
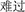
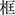
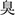
Expectedly			Uneasy	Embryo			Betray
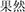
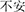
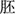
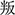
Actually			Furious	Crop			Wither
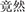
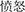
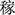
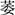
Have to			Sad	Glue			Inferior
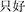
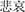
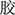
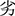
Virtually			Disappointed	Vertical			Mildew
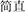
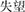
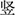
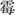
Never			Angry	Increase			Disaster
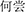
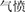
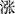
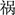
However			Fickle	Freeze			Greedy
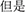
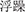
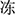
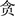
Because			Spiritless	Complete			Dirty
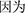
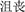
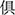
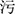
If			Distressed	Ginger			Bother
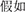
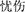
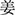
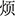
Seemingly			Despondent	Fence			Grave
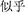
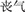
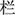
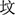
Although			Disappointed	Green			Tumor
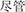
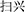
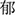
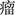
Although			Discouraged	Seedling			Rot
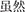
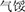
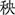
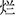
Even if			Listless	Basket			Mourning
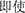
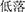
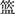
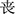
Previously			Jealous	Swim			Robber
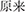
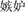
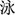
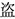
Besides			Disturbing	Old man			Foxy
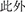
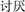
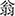
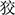
Alike			Boring	Jar			Destroy
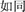
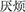
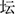
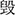
Ever			Depressive	Boiling			Collapse
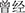
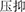
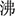
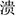
Because			Wronged	Swell			Tragic
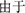
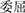
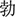
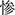
Really			Panic	Cabinet			Funeral
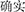
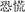
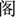
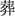
Supposing			Ashamed	Female			Fraud
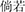
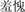
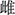
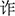
Again			Shameful	Care			Thief
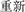
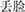
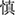
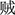
Consistently			Disgraceful	Expect			Abhor
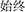
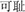
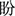
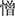
Finally			Indifferent	Hot			Blind
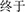
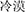
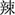
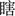
Almost			Low spirited	Pale			Decadent
